# Antimicrobial resistance and genomic investigation of non-typhoidal *Salmonella* isolated from outpatients in Shaoxing city, China

**DOI:** 10.3389/fpubh.2022.988317

**Published:** 2022-09-13

**Authors:** Jiancai Chen, Abdelaziz Ed-Dra, Haiyang Zhou, Beibei Wu, Yunyi Zhang, Min Yue

**Affiliations:** ^1^Zhejiang Provincial Center for Disease Control and Prevention, Hangzhou, China; ^2^Hainan Institute of Zhejiang University, Sanya, China; ^3^Department of Veterinary Medicine, Institute of Preventive Veterinary Sciences, Zhejiang University College of Animal Sciences, Hangzhou, China; ^4^Zhejiang Provincial Key Laboratory of Preventive Veterinary Medicine, Hangzhou, China; ^5^State Key Laboratory for Diagnosis and Treatment of Infectious Diseases, National Clinical Research Center for Infectious Diseases, National Medical Center for Infectious Diseases, The First Affiliated Hospital, College of Medicine, Zhejiang University, Hangzhou, China

**Keywords:** non-typhoidal *Salmonella*, whole genome sequencing, antimicrobial resistance, virulence, gastroenteritis, public health, salmonellosis

## Abstract

Human non-typhoidal salmonellosis is among the leading cause of morbidity and mortality worldwide, resulting in huge economic losses and threatening the public health systems. To date, epidemiological characteristics of non-typhoidal *Salmonella* (NTS) implicated in human salmonellosis in China are still obscure. Herein, we investigate the antimicrobial resistance and genomic features of NTS isolated from outpatients in Shaoxing city in 2020. Eighty-seven *Salmonella* isolates were recovered and tested against 28 different antimicrobial agents, representing 12 categories. The results showed high resistance to cefazolin (86.21%), streptomycin (81.61%), ampicillin (77.01%), ampicillin-sulbactam (74.71%), doxycycline (72.41%), tetracycline (71.26%), and levofloxacin (70.11%). Moreover, 83.91% of isolates were resistant to ≥3 categories, which were considered multi-drug resistant (MDR). Whole-genome sequencing (WGS) combined with bioinformatic analysis was used to predict serovars, MLST types, plasmid replicons, antimicrobial resistance genes, and virulence genes, in addition to the construction of phylogenomic to determine the epidemiological relatedness between isolates. Fifteen serovars and 16 STs were identified, with the dominance of *S*. I 4, [5], 12:i:– ST34 (25.29%), *S*. Enteritidis ST11 (22.99%), and *S*. Typhimurium ST19. Additionally, 50 resistance genes representing ten categories were detected with a high prevalence of *aac(6')-Iaa* (100%), *bla*_TEM−1B_ (65.52%), and *tet(A)* (52.87%), encoding resistance to aminoglycosides, β-lactams, and tetracyclines, respectively; in addition to chromosomic mutations affecting *gyrA* gene. Moreover, we showed the detection of 18 different plasmids with the dominance of IncFIB(S) and IncFII(S) (39.08%). Interestingly, all isolates harbor the typical virulence genes implicated in the virulence mechanisms of *Salmonella*, while one isolate of *S*. Jangwani contains the *cdtB* gene encoding typhoid toxin production. Furthermore, the phylogenomic analysis showed that all isolates of the same serovar are very close to each other and clustered together in the same clade. Together, we showed a high incidence of MDR among the studied isolates which is alarming for public health services and is a major threat to the currently available treatments to deal with human salmonellosis; hence, efforts should be gathered to further introduce WGS in routinely monitoring of AMR *Salmonella* in the medical field in order to enhance the effectiveness of surveillance systems and to limit the spread of MDR clones.

## Introduction

Gastroenteritis is a common disease in both developing and developed countries and is considered a significant economic burden leading to high financial loss for worldwide health care systems. Most gastroenteritis cases are self-limited in immunocompetent patients. At the same time, it can persist for a long time with severe symptoms and diarrhea in immunocompromised patients, including young children and the elderly. According to recent data, over 1.7 billion global cases of diarrheal disease are reported annually, leading to about 2.2 million deaths (1). In China, infectious diarrhea, excluding cholera, dysentery, and enteric fever, caused more than one million cases annually from 2014 to 2019, which the National Notifiable Diseases Reporting System classified as Category C infectious disease (2). Bacteria take second place among the agents causing gastroenteritis after viruses (3).

Non-typhoidal *Salmonella* (NTS) are among the most common etiological agents causing acute gastroenteritis worldwide. It is estimated that *Salmonella* species were responsible for about 180 million (9%) of the diarrheal diseases that occur globally each year, leading to 298,000 deaths, representing 41% of all diarrheal disease-associated deaths (4, 5). In China, the incidence of non-typhoidal salmonellosis was estimated at 626.5 cases per 100,000 persons (6). *Salmonella* is a Gram-negative, rod-shaped, non-spore-forming, and facultatively anaerobic bacterium, belonging to the Enterobacteriaceae family. To date, more than 2,600 *Salmonella* serovars have been described (7), where only some of them were reported to cause human salmonellosis (8–17). The digestive tract of vertebrates is considered the main reservoir of *Salmonella* species, and animal farms are the primary source for the development and dissemination (6, 18–22). *Salmonella* might contaminate animal carcasses during transport, slaughtering, and then transmitted to humans *via* the farm-to-fork route (23–30), causing severe infections and threatening public health systems.

Antibiotics have been widely used to treat salmonellosis in the veterinary or medical fields. However, since the 1950s, resistance to the usual antimicrobial agents has appeared and increased until rising the threatened limits. Currently, the third-generation cephalosporins and quinolones are used as the last line of defense to treat salmonellosis in both adult and young patients, while polymyxins are used to treat the cases of multidrug resistance Enterobacteriaceae (31, 32). However, recent investigations have reported the resistance of *Salmonella* isolates recovered from animal farms, food chain processes, foods, and humans to different antibiotics, including the third-generation cephalosporins, quinolones, polymyxins, aminoglycosides, and others (18, 31, 33), with the usual detection of superbug isolates which further complicates the epidemiological situation and is considered a serious threat to public health by limiting choices for therapeutic treatment of patients (34).

In this regard, the earlier detection and accurate diagnosis of multidrug-resistant (MDR) isolate based on high throughput surveillance are the key solutions to limit the spread and dissemination of harmful superbug clones. Today the advance in high throughput sequencing encourages the use of WGS on a large scale for the epidemiological investigation to monitor MDR pathogens (23, 35). Additionally, the ongoing decreases in sequencing costs and the increase of online platforms available for analyzing, sharing, and comparing genomic data, enormously helped the harmonization use of WGS in different areas (36–38). In China, several cases of human salmonellosis were recorded each year, however, the in-depth analysis of the implicated *Salmonella* isolates is still obscure. To proof of concept, this study aims to use WGS as a cost-effective method to provide genomic features, including MLST patterns, antimicrobial resistance and virulence genes, plasmid replicons, and genetic diversity of *Salmonella* isolates recovered from outpatients in Shaoxing city, Zhejiang province, China.

## Materials and methods

### Sample collection and *Salmonella* identification

A total of 87 *Salmonella* isolates were collected from outpatients in different counties (Zhuji, Shengzhou, Xinchang, Keqiao, Shangyu, and Yuecheng) of Shaoxing city, Zhejiang province, China (Supplementary Table S1). During the year 2020, diarrheal samples were collected from outpatients having gastroenteritis. *Salmonella* isolates were isolated, identified, and characterized according to the previously described methods (11, 12, 14, 16). Briefly, a 10 mL buffered peptone water was used for the pre-enrichment of human fecal samples (Oxoid, United Kingdom), then 0.1 mL of the pre-enriched samples were added to 10 mL of Rappaport Vassiliadis broth (Oxoid, United Kingdom) and incubated at 42°C for 24 h. After incubation, the samples were streaked onto Xylose Lysine Desoxycholate (XLD) (Oxoid, United Kingdom) and incubated at 37°C for 18–24 h. The suspected colonies of *Salmonella* on XLD were round, transparent red or pink with or without typical black centers. The suspected colonies were confirmed using matrix-assisted laser desorption ionization-time of flight mass spectrometry (MALDI-TOF MS) and polymerase chain reaction (PCR) to amplify the *invA* gene using specific primers as described previously (5, 16, 39). Furthermore, The PCR-confirmed *Salmonella* isolates were serotyped by slide agglutination method to define O and H antigens using commercial antisera (SSI Diagnostica, Hillerød, Denmark) according to White–Kauffmann–Le Minor scheme (40).

### Antimicrobial susceptibility testing

The antimicrobial susceptibility of the studied *Salmonella* isolates was evaluated toward a panel of 28 different antimicrobial agents belonging to 12 categories by using the broth dilution method. The tested antimicrobial agents were as follow: Penicillins (Ampicillin, AMP), β-lactam combination agents (Amoxicillin-clavulanic acid, AMC; Ampicillin-sulbactam, SAM), Aminoglycosides (Amikacin, AMK; Gentamicin, GEN; Kanamycin, KAN; Streptomycin, STR), Tetracyclines (Tetracycline, TET; Minocycline, MIN; Doxycycline, DOX); Phenicols (Chloramphenicol, CHL), Folate pathway inhibitors (Trimethoprim-sulfamethoxazole, SXT; Sulfafurazole, SIZ); Cephems (Cefazolin, CFZ; Cefoxitin, FOX; Cefotaxime, CTX; Ceftazidime, CAZ; Cefepime, FEP); Carbapenems (Imipenem, IPM; Meropenem, MEM); Quinolones (Nalidixic acid, NAL; Ciprofloxacin, CIP; levofloxacin, LVX; Gemifloxacin, GEM); Macrolides (Azithromycin, AZM); Lipopeptides (Colistin, CST; Polymyxin B, PMB); Monobactams (Aztreonam, ATM) (Table 1). The interpretation of results was performed according to the recommendation of the Clinical Laboratory Standard Institute guidelines (CLSI), and the European Committee for Antimicrobial Susceptibility Testing (EUCAST) (41, 42), and the isolates presented intermediate resistance were considered resistant for the ease of analysis. However, isolates that are non-susceptible to at least one antimicrobial agent in three or more than three antimicrobial categories were considered multidrug-resistant (MDR) (43). *Escherichia coli* ATCC 25922 was used as a control strain.

**Table 1:** Antimicrobial susceptibility percentage of *Salmonella* (*n* = 87) isolated from outpatients.

**Antimicrobial agent**	**Code**	**Concentration range (μg/ mL)**	**Breakpoint interpretive criteria (**μg/mL)^*^			**Results in percentage (%)**	
			**S**	**I**	**R**	**S**	R^**^
**Aminoglycosides**							
Amikacin	AMK	0.5–128	≤16	32	≥64	87 (100%)	0 (0%)
Gentamicin	GEN	0.12–128	≤4	8	≥16	77 (88.51%)	10 (11.49%)
Kanamycin	KAN	0.5–128	≤16	32	≥64	82 (94.25%)	5 (5.75%)
Streptomycin^a^	STR	0.5–128	≤8	16	≥32	16 (18.39%)	71 (81.61%)
**β-lactam combination agents**							
Amoxicillin-clavulanic acid	AMC	0.25/0.12–128/64	≤8/4	16/8	≥ 32/16	65 (74.71%)	22 (25.29%)
Ampicillin-sulbactam	SAM	0.25/0.12–128/64	≤8/4	16/8	≥ 32/16	22 (25.29%)	65 (74.71%)
**Tetracyclines**							
Tetracycline	TET	0.12–128	≤4	8	≥16	25 (28.74%)	62 (71.26%)
Minocycline	MIN	0.12–128	≤4	8	≥16	40 (45.98%)	47 (54.02%)
Doxycycline	DOX	0.12–128	≤4	8	≥16	24 (27.59%)	63 (72.41%)
**Folate pathway inhibitors**							
Trimethoprim-sulfamethoxazole	SXT	0.25/4.75–32/608	≤2/38	–	≥4/76	63 (72.41%)	24 (27.59%)
Sulfafurazole^b^	SIZ	16–512	≤256	–	≥512	32 (36.78%)	55 (63.22%)
**Cephems**							
Cefazolin	CFZ	0.032–64	≤2	4	≥8	12 (13.79%)	75 (86.21%)
Cefoxitin	FOX	0.5–128	≤8	16	≥32	87 (100%)	0 (0%)
Cefotaxime	CTX	0.032–64	≤1	2	≥4	78 (89.66%)	9 (10.34%)
Ceftazidime	CAZ	0.032–64	≤4	8	≥16	81 (93.10%)	6 (6.90%)
Cefepime	FEP	0.032–64	≤2	–	≥16	85 (97.70%)	2 (2.30%)
**Carbapenems**							
Imipenem	IPM	0.12–64	≤1	2	≥4	86 (98.85%)	1 (1.15%)
Meropenem	MEM	0.12–64	≤1	2	≥4	86 (98.85%)	1 (1.15%)
**Quinolones**							
Nalidixic acid	NAL	0.5–128	≤16	–	≥32	51 (58.62%)	36 (41.38%)
Ciprofloxacin	CIP	0.004–16	≤0.06	0.12~0.5	≥1	28 (32.18%)	59 (67.82%)
Levofloxacin	LVX	0.004–16	≤0.12	0.25~1	≥2	26 (29.89%)	61 (70.11%)
Gemifloxacin	GEM	0.004–16	≤0.25	0.5	≥1	72 (82.76%)	15 (17.24%)
**Macrolides**							
Azithromycin	AZM	0.5–128	≤16	–	≥32	81 (93.10%)	6 (6.90%)
**Lipopeptides**							
Colistin	CST	0.12–32	≤2	–	≥4	67 (77.01%)	20 (22.99%)
Polymyxin B	PMB	0.12–32	≤2	–	≥4	73 (83.91%)	14 (16.09%)
**Monobactams**							
Aztreonam	ATM	0.25–64	≤4	8	≥16	79 (90.80%)	8 (9.20%)
**Penicillins**							
Ampicillin	AMP	0.5–64	≤8	16	≥32	20 (22.99%)	67 (77.01%)
**Phenicols**							
Chloramphenicol	CHL	0.5–128	≤8	16	≥32	59 (67.82%)	28 (32.18%)

* **S**, susceptible; **I**, intermediate resistance; **R**, resistant.

** Intermediate results were merged with resistant results.

a For streptomycin, we used the same MIC breakpoints as for netilmicin.

b For Sulfafurazole, we used the same MIC breakpoints as for sulfonamides.

### Genomic DNA extraction and sequencing

The genomic DNA of all the studied isolates were extracted using TIANamp bacteria DNA kit (Tiangen Biotech, China) according to the manufacturer's instructions, from overnight cultures in Luria–Bertani (LB) broth incubated at 37 °C. The obtained genomic DNA was quantified using a Qubit 2.0 fluorometer (Invitrogen, USA) and then sequenced by using Illumina NovaSeq 6000 platform as previously described (14, 20, 35).

### Bioinformatic analysis

The obtained raw reads were checked for sequencing quality using FastQC and trimmed using Trimmomatic (44), in which the low-quality sequences and joint sequences were removed. The clean data were then assembled using SPAdes 4.0.1 (45) with “careful” correction option to reduce the number of mismatches in the final assembly and annotated by using the Rapid Annotation Subsystem Technology (RAST) server (https://rast.nmpdr.org/) and Prokka v.1.14 (46). The assembled contigs were then used to predict plasmid replicons and antimicrobial-resistance genes using PlasmidFinder 2.1 (https://cge.cbs.dtu.dk/services/PlasmidFinder/) and ResFinder 3.2 tools (https://cge.cbs.dtu.dk/services/ResFinder/), respectively, with a similarity cut-off of 90%. The prediction of serovar and sequence type were performed using SeqSero 1.2 (https://cge.food.dtu.dk/services/SeqSero/) and MLST 2.0 (https://cge.food.dtu.dk/services/MLST/) available on the Center for Genomic Epidemiology (CGE) platform. The genomic mutations affecting the quinolone resistance-determining region (QRDR) were detected by using Staramr software against PointFinder v1.9 (https://github.com/phac-nml/staramr). Additionally, virulence genes were predicted using the virulence factors database (VFDB) (47). All bioinformatic analyses were conducted on our in-house Galaxy platform as previously described (20). On the other hand, the association between antimicrobial resistance genes and the corresponding plasmid replicons has been investigated according to the method described previously (18).

### Phylogenomic analysis

The core single-nucleotide polymorphism (SNP) of all the studied *Salmonella* strains was analyzed using Snippy v4.4.4 against the reference strain *S*. Typhimurium SL 1344. The phylogenetic tree was constructed using IQ-TREE v.1.6.12 with TVM + F + ASC model and 1,000 bootstraps, as previously described (13, 22). Moreover, the whole-genome multilocus sequence typing (wgMLST) was used to conduct wgMLST phylogenomic tree by using cano-wgMLST_Bac Compare software as described recently (20, 48), with default parameters and *S*. Typhimurium strain SL 1344 as reference strain.

## Results

### Distribution of *Salmonella* serovars

**Figure 1:**
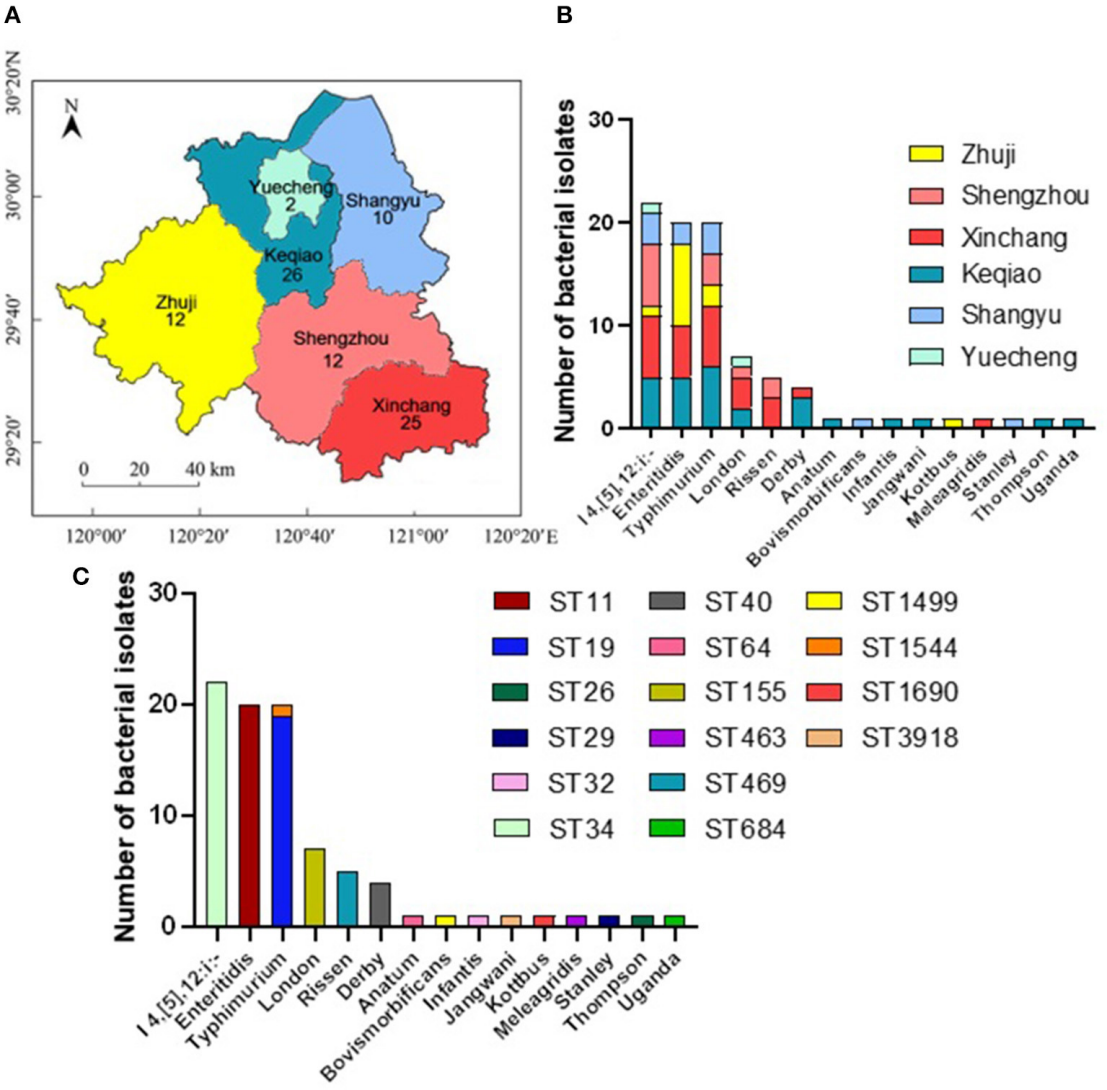
The distribution of different serovars among six counties in Shaoxing city, Zhejiang province, China. **(A)** The geographical distribution of the *Salmonella* isolates in Shaoxing with six counties which were examined in current investigations. N.B., The number indicates the numbers of isolates collected from individual county. **(B)** The distribution of fifteen serovars of *Salmonella* isolates. The dominant serovars are *S*. I 4, [5], 12:i:-, *S*. Enteritidis, and *S*. Typhimurium. **(C)** The prevalence of individual serovar with their sequence type (ST) detected in this study.

In this study, 87 *Salmonella* isolates have been isolated and identified, including 26 from Keqiao county (29.88%), 25 from Xinchang (28.74%), 12 from Zhuji (13.79%), 12 from Shengzhou (13.79%), ten from Shangyu (11.49%), and two from Yuecheng (2.30%) (Figure 1A). These isolates have been shared between male (44/87; 50.57%) and female (49.43%) patients (Supplementary Table S1). The serotyping method has identified 15 different serovars with the dominance of the monophasic variant I 4, [5], 12:i:– (22/87; 25.29%), followed by Enteritidis (20/87; 22.99%), and Typhimurium (Figure 1B). Additionally, allelic profiles analysis showed the identification of 16 sequence types (STs) with the dominance of ST34 (22/87; 25.29%), ST11 (20/87; 22.99%), and ST19 (19/87; 21.84%) (Figure 1C). All serovars presented one ST, except *S*. Typhimurium, which was divided into ST19 (*n* = 19) and ST1544 (*n* = 1).

### Phenotypic antimicrobial resistance

**Figure 2:**
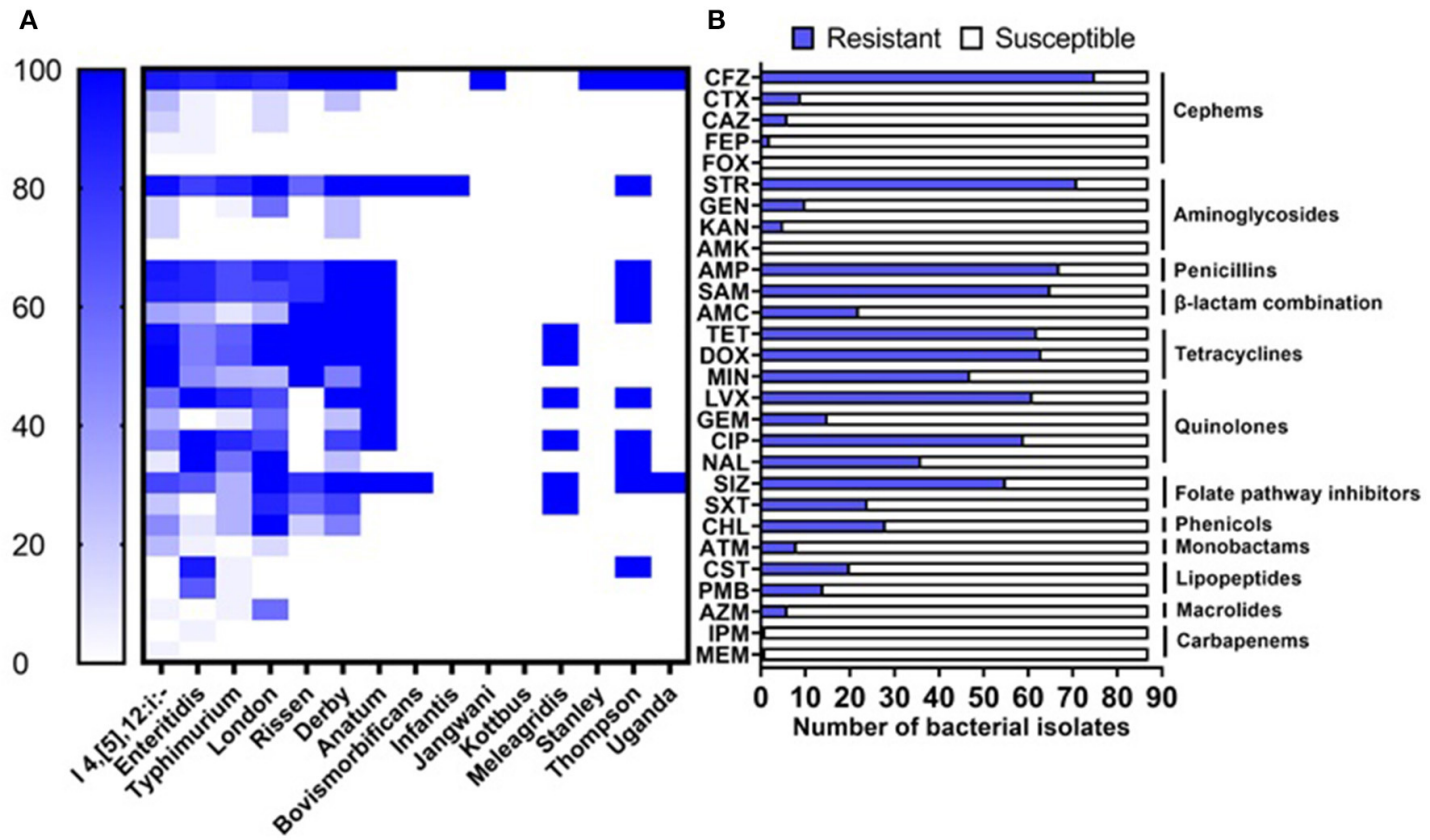
Antimicrobial resistance of *Salmonella* isolates. **(A)** Resistance of isolates grouped by serovars to the tested antimicrobial agents. **(B)** Prevalence of resistance against 28 antimicrobial agents belonging to 12 categories.

Phenotypic antimicrobial susceptibility of the 87 isolates has been evaluated toward 28 antimicrobial agents representing 12 different categories by broth dilution method according to CLSI and EUCAST recommendations. The highest resistance was observed against cefazolin (75/87; 86.21%), streptomycin (71/87; 81.61%), ampicillin (67/87; 77.01%), the combination of ampicillin-sulbactam (65/87; 74.71%), doxycycline (63/87; 72.41%), tetracycline (62/87; 71.26%), and levofloxacin (61/87; 70.11%), while all isolates were susceptible to cefoxitin and amikacin (Table 1, Figure 2B). Based on serovars distribution, our results showed that the abundant serovars (>3 isolates), especially the monophasic variant I 4, [5], 12:i:–, Enteritidis, Typhimurium, London, and Derby, present high resistance to multiple antimicrobial agents (Figure 2A). Additionally, we detected 57 different antimicrobial resistance patterns, in which 85/87 (97.70%) of isolates presented resistance to at least one category, 73/87 (83.91%) given resistance to three or more than three categories which were considered MDR. In contrast, 67/87 (77.01) isolates were resistant to five or more antimicrobial categories (Supplementary Table S2).

### Genotypic antimicrobial resistance

**Figure 3:**
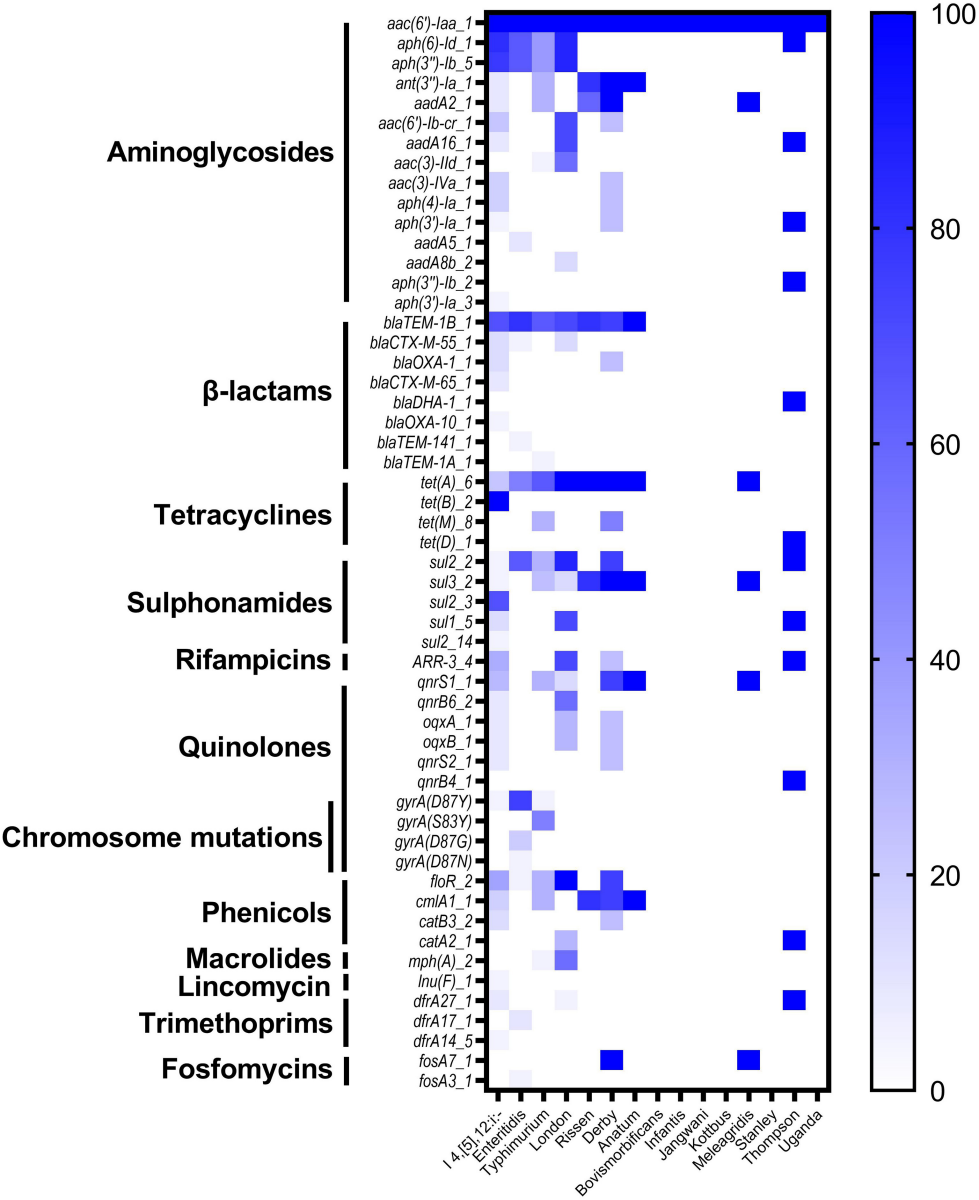
The heatmap of antimicrobial resistance genes (ARGs) and chromosomic mutations in the studied *Salmonella* isolates.

In order to understand the genetic arsenal behind the acquisition of antimicrobial resistance. The whole genome sequences of all isolates (*n* = 87) were screened for the detection of antimicrobial determinants encoding resistance to different antimicrobial categories, in addition to genomic mutations in the quinolone resistance-determining region (QRDR) affecting the DNA gyrase and DNA topoisomerase IV genes. The results showed the detection of 50 determinants encoding resistance to 11 different antimicrobial categories, in addition to four different mutations on the gene *gyrA* encoding resistance to quinolone (Supplementary Table S1). The most prevalent antimicrobial determinants were *aac (6')-Iaa_1* (100%), *aph (6)-Id_1* (52.87%), and *aph (3”)-Ib_5* (50.57%) encoding resistance to aminoglycosides, *bla*_TEM−1B_1_ (65.52%) encoding resistance to β-lactams, and *tet (A)_6* (52.87%) encoding resistance to tetracyclines (Supplementary Table S1). The monophasic variant of *S*. Typhimurium I 4, [5], 12:i:– harbors more diversified resistance genes (36 genes) followed by serovars London and Derby (22 genes for each one), while serovars Typhimurium and Enteritidis harbor 18 and 15 resistance genes, respectively (Figure 3). Additionally, genomic mutations conferring resistance to quinolones were only detected in serovars I 4, [5], 12:i:–, Enteritidis, and Typhimurium, where single and double mutations in the gene *gyrA* were observed (Figure 3).

### Virulence genes prediction

**Figure 4:**
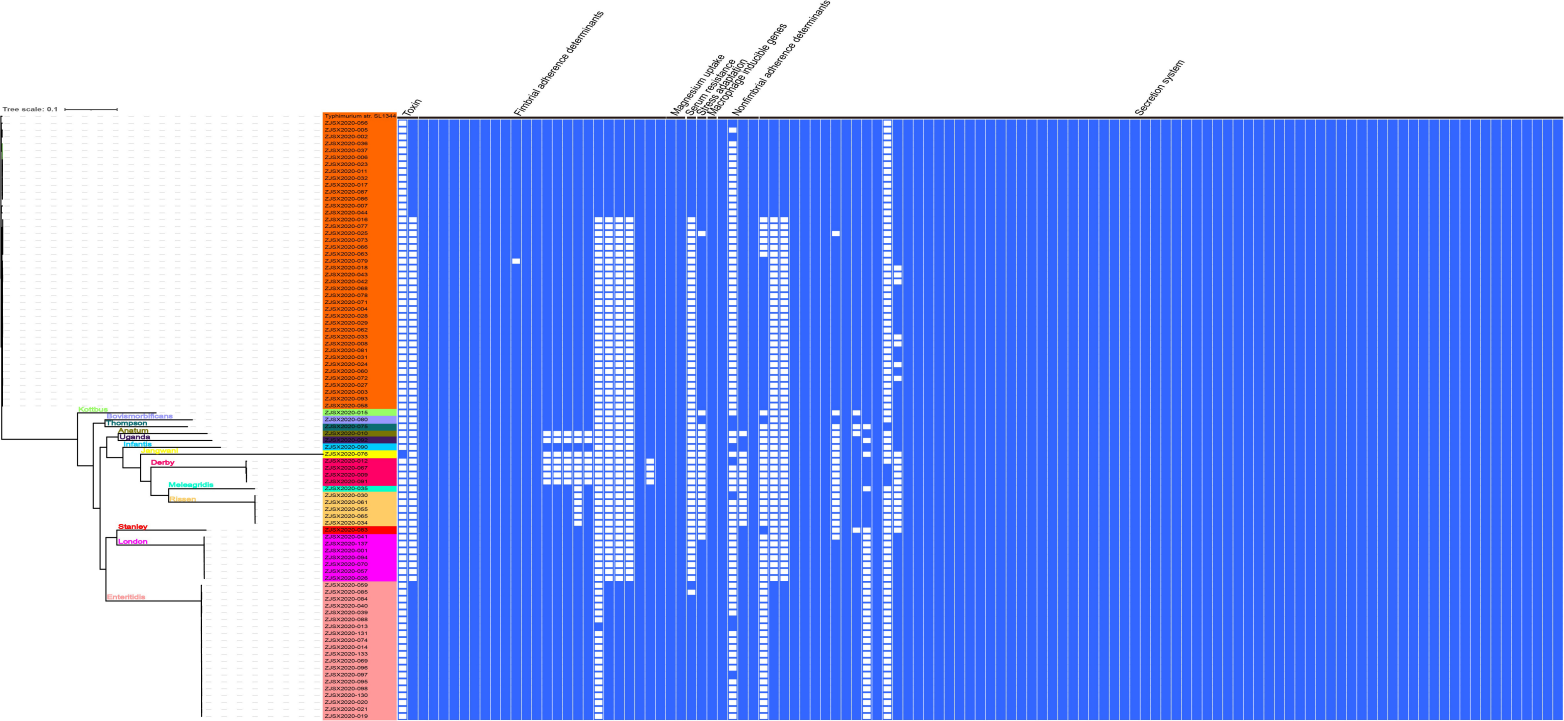
The combinatorial graph of the wgMLST phylogenomic evolutionary tree and virulence genes in the studied *Salmonella* isolates. The presence of virulence gene was marked with blue color while the absence was marked with white color. The tree was rooted by using *S*. Typhimurium SL 1344.

To provide accurate data about the virulence of studied isolates, we conducted an in-depth analysis to predict virulence genes implicated in the virulence and pathogenicity mechanism of *Salmonella* based on WGS, and the results were presented in Figure 4 and Supplementary Table S1. We detected 117 different virulence genes and the number of genes per isolate varies between 92 and 112. In addition to the typical virulence genes carried on *Salmonella* pathogenicity islands (SPIs), we detected *cdtB* gene encoding typhoid toxin production in one isolate of *S*. Jangwani. The *spv* locus that clustered genes implicated in the virulence system of non-typhoid *Salmonella*, the *pef* locus clustered genes encoding fimbriae, and *rck* gene encoding serum resistance, were only detected in *Salmonella* serovars Typhimurium and Enteritidis. Furthermore, *sodCI* gene encoding for stress adaptation was detected in the monophasic variant I 4, [5], 12:i:–, *S*. Typhimurium, *S*. Enteritidis, *S*. London, and *S*. Bovismorbificans. While the gene *grvA* encoding for anti-virulence was detected in serovars London, Typhimurium, and Bovismorbificans.

### Plasmid replicons

**Figure 5:**
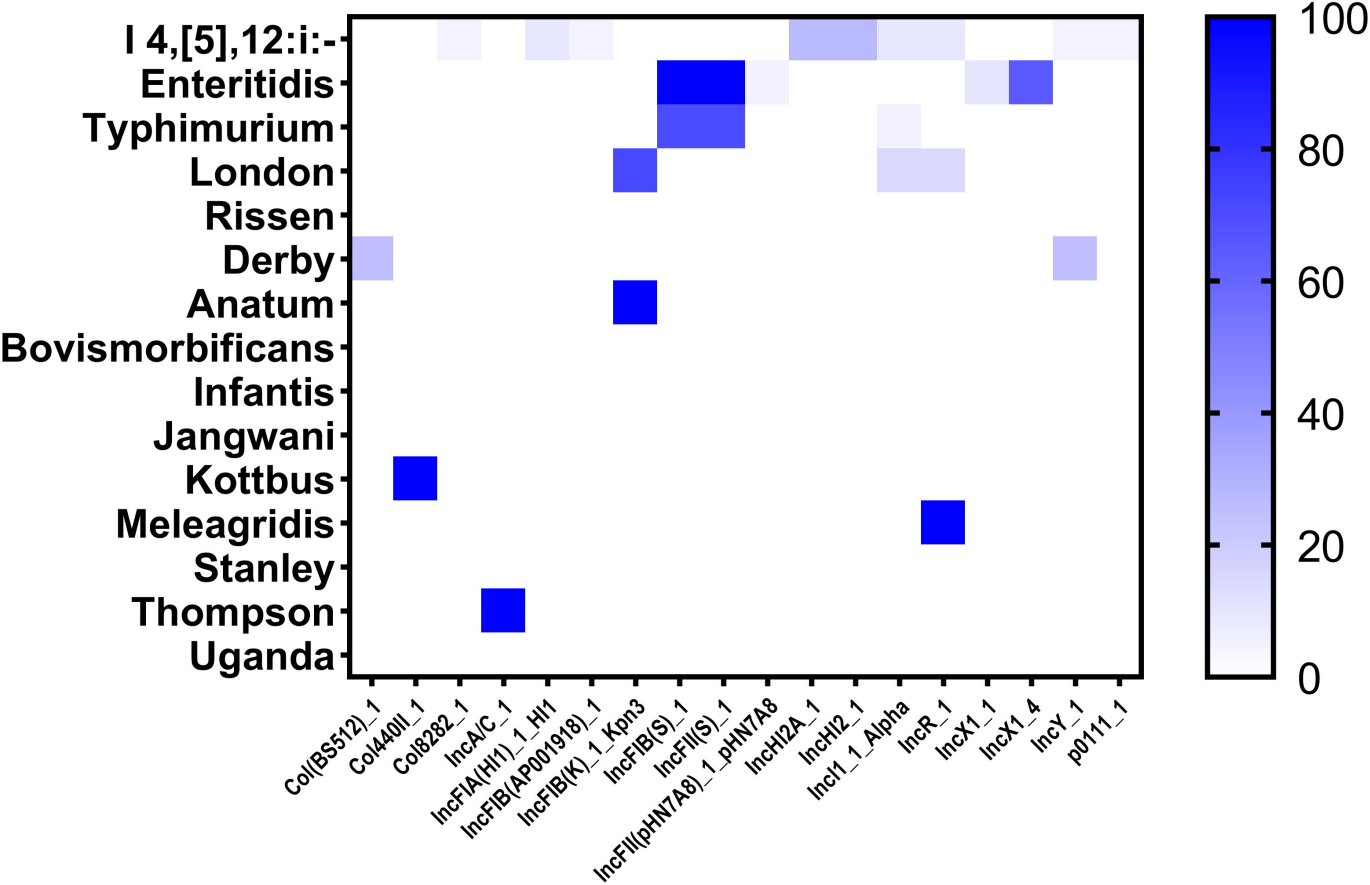
The heatmap of plasmids distribution in different *Salmonella* isolates. The strength of the colors corresponds to the numerical value of the prevalence of the plasmids. Dark blue color indicates high prevalence and white color for gene absence.

The distribution of plasmid replicons among the studied *Salmonella* isolates was performed by screening the whole genome sequences in the PlasmidFinder tool. In this study, 18 different plasmids were detected where the plasmids IncFIB (S)_1 and IncFII (S)_1 were the most prevalent (34/87; 39.08% for each one), followed by IncX1_4 (13/87; 14.94%), and IncHI2A_1, IncHI2_1 and IncFIB(K)_1_Kpn3 (6/87; 6.90% for each one) (Supplementary Table S1). Additionally, we reported that the monophasic variant I 4, [5], 12:i:– harbors more diversified plasmids (*n* = 9) compared with other serovars, followed by Enteritidis (*n* = 5); However, isolates belonging to serovars Rissen, Bovismorbificans, Infantis, Jangwani, Stanley, and Uganda didn't harbor any plasmid (Figure 5).

### Association of plasmids and antimicrobial resistance genes

The association between plasmid replicons and antimicrobial resistance genes has been investigated and the results are presented in Supplementary Table S3. We demonstrated that the plasmid type IncX1 was the main carrier of antimicrobial resistance, carrying multiple resistance genes like *sul2, aph (3”)-Ib, aph (6)-Id, tet (A)* encoding resistance to sulphonamides, aminoglycosides, and tetracyclines, respectively. However, the plasmid types IncFII(S) and IncFIB(S) were the leading carriers of the gene *bla*_TEM−1B_ encoding resistance to β-lactam. Notably, the co-occurrence between plasmid replicons and antimicrobial resistance genes was mainly observed in serovar Enteritidis.

### Phylogenomic analysis

**Figure 6:**
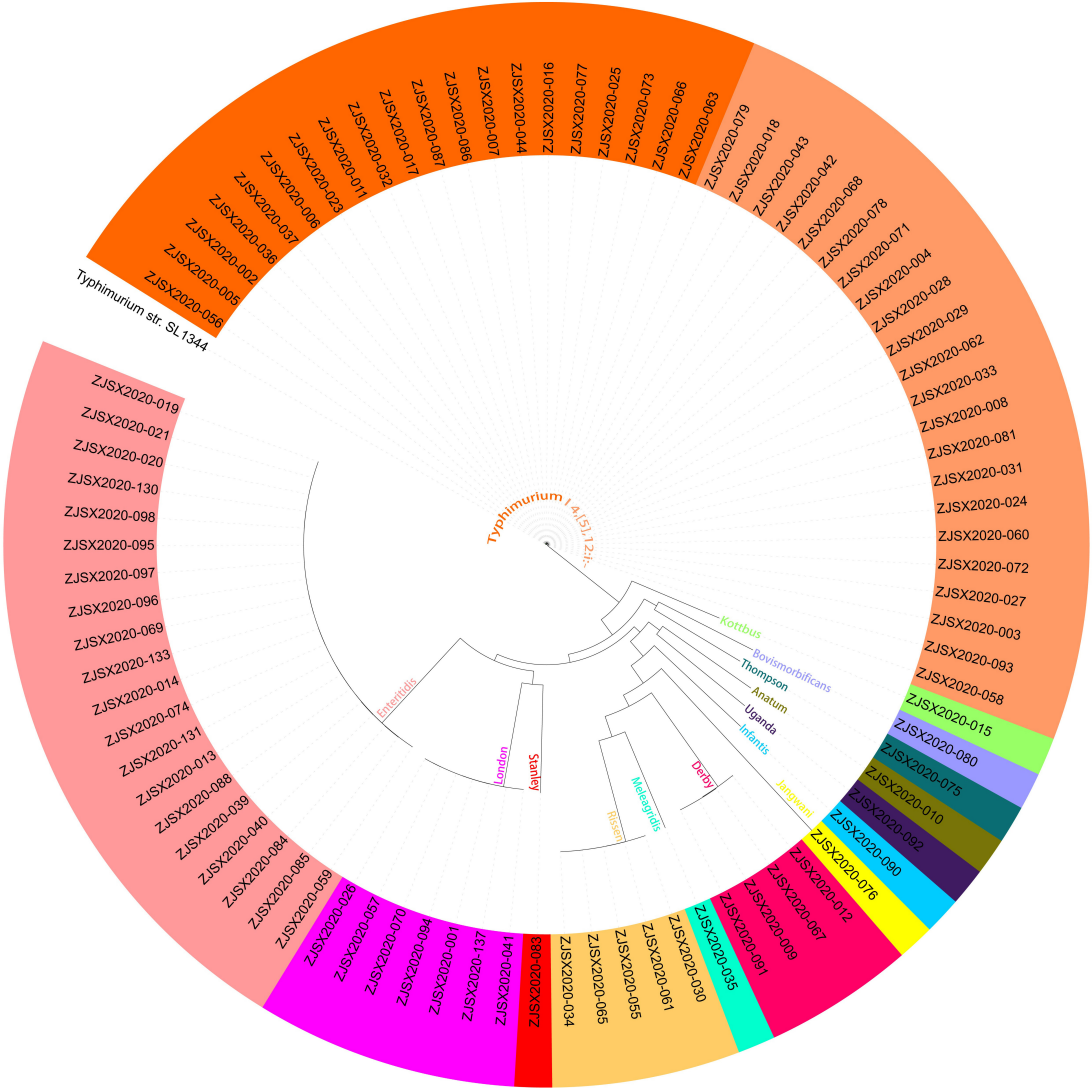
The core genome SNP-based phylogenomic tree by maximum likelihood (TVM+F+ASC) of 87 *Salmonella* isolates. The tree was rooted by using S. Typhimurium SL 1344.

The sequenced and assembled genomes have been first checked for quality assessment and results were presented in Supplementary Table S4. The results showed that the number of contigs (≥300 bp) varies between 30 and 117 contigs while the average genomes size of draft assemblies was 4,920,864 bp and the average N_50_ was 320,121 bp. Moreover, to determine the epidemiological relatedness between the studied *Salmonella* isolates, we conducted a phylogenomic analysis based on core single-nucleotide polymorphism (SNP) and whole-genome multilocus sequence typing (wgMLST) (Figure 4, 6). The results showed that isolates belonging to the same serovar are very close to each other and clustered together in the same clade. A substantial similarity has been seen between isolates of the monophasic variant I 4, [5], 12:i: and Typhimurium, which were grouped in the same clade. Moreover, isolates of serovars Enteritidis, London, and Stanley have been grouped in the same clade indicating a high association between them. Furthermore, the association between phylogenomic tree and distribution of virulence factors showed few differences in the patterns of virulence factor cassettes between different serovars (Figure 4).

## Discussion

Non-typhoidal salmonellosis is a common disease caused by the bacteria *Salmonella*, leading to a huge economic burden, which threatens healthcare systems worldwide (49–51). *Salmonella* originated from animal reservoirs are transmitted to humans *via* the food chain causing gastroenteritis and sometimes invasive infections. Generally, the consumption of food or water contaminated with NTS is the leading cause of human salmonellosis. In this study, we presented the epidemiological characteristics of NTS isolates recovered from outpatients in Shaoxing city based on WGS combined with accurate bioinformatics tools and in-depth phenotypic methods, these results may provide the scientific background about the MDR *Salmonella* circulated in Chinese hospitals and may help health service authorities to implement effective programs to limit the spread and dissemination of MDR clones in medical field.

In this study, 87 *Salmonella* isolates were identified, these isolates belonged to 15 different serovars and 16 STs. The dominant serovar was the monophasic variant I 4, [5], 12:i:– ST34, followed by *S*. Enteritidis ST11, and *S*. Typhimurium ST19. These serovars are currently considered the most common serovars implicated in human salmonellosis (52, 53). Since the mid-1990s, the implication of the monophasic variant I 4, [5], 12:i:– in human salmonellosis has significantly increased, which is currently classified among the most five predominant serovars in NTS infections (52, 54). However, *S*. Enteritidis is well known as a worldwide foodborne pathogen and is widely linked to the consumption of egg-derived products (55, 56). In China, *S*. Typhimurium is considered the most common serovar causing NTS infections. Ke et al. reported that *S*. Typhimurium was the most predominant serovar (62.6%) among isolates recovered from children between 2012 and 2019 in a tertiary hospital in Ningbo, Zhejiang, China (57). Similarly, Wu et al. showed the dominance of *S*. Typhimurium (79.2%) among isolates recovered from children with NTS infections between 2009 and 2018 in Chongqing, China (58). Another Chinese study carried out in the Conghua district of Guangzhou, between June and October 2020 isolated *S*. Typhimurium as the most common serovar in hospitalized patients (59). Moreover, in Vietnam *S*. Typhimurium dominated NTS isolates (41.8%) from hospitalized children in Ho Chi Minh city, Vietnam (60). In Thailand, Sinwat et al. reported *S*. Typhimurium as the major serovar for diarrhoeal patients (61). The wide spread of *S*. Typhimurium among patients in Southeast Asian countries could be due to the common source of contamination, especially with the high trading exchange between these countries.

In order to provide accurate epidemiological information about the current antimicrobial susceptibility situation of clinical NTS, all the isolated strains were tested against a panel of 28 different antimicrobial agents belonging to 12 categories. The common resistance was observed against cefazolin (1st generation of cephalosporin), streptomycin (aminoglycoside), ampicillin (penecillins), the combination of ampicillin-sulbactam, doxycycline and tetracycline (tetracyclines), and levofloxacin (quinolones). The high resistance to these categories has becoming frequently observed in *Salmonella* isolates recovered from food, human, and animal samples (6, 19, 25, 26, 58, 62). Moreover, we reported a high incidence of MDR among isolates (83.91%) which was higher than that found in *Salmonella* recovered from hospitalized patients in the Conghua district of Guangzhou, China (47.06%) (59), and from children with NTS infections in Chongqing, China (13.7%) (58). Indeed, the extensive and continued use of antibiotics in medical and veterinary fields has led to the development of MDR strains which threaten public health by limiting the effectiveness of available antimicrobials for therapeutic usage. Hence, infections with these MDR isolates may lead to therapeutic failure, especially with the limit in alternative antibiotics able to treat these cases.

The screening of antimicrobial resistance genes was performed based on WGS and results showed the detection of 50 genes encoding resistance to 11 categories in addition to 4 mutations affecting the gene *gyrA*. All isolates (100%) were positive for the genes *aac (6')-Iaa* encoding resistance to aminoglycosides, while 65.52% contain *bla*_TEM−1B_ encoding resistance to β-lactams and 52.87% contain *tet (A)* gene encoding resistance to tetracyclines. Generally, resistance to aminoglycosides is associated with enzymatic modification by using certain enzymes, including aminoglycoside phosphotransferases, aminoglycoside acetyltransferases, and aminoglycoside adenyltransferases encoded by the genes *aphA, aacC*, and *aadA*, respectively (63, 64). Resistance to β-lactams is mediated by *bla* genes encoding enzymes (β-lactamases) capable of hydrolyzing the β-lactam ring of β-lactam antibiotics. In this study, we detected *bla* genes in predominant serovars, including the monophasic variant I 4, [5], 12:i:–, Enteritidis, Typhimurium, in addition to serovars London, Rissen, Derby, Anatum, and Thompson. Thus, it has been reported that different types of *bla*_TEM_ are mostly linked with ampicillin-resistant *Salmonella* serovars (65), which may explain the high incidence of ampicillin resistance. We also detected the *tet* genes encoding resistance to tetracyclines, in addition to different genes encoding resistance to chloramphenicol. Resistance to tetracyclines and chloramphenicol is often associated with efflux pumps mechanisms encoded by *tet* and *floR* genes, respectively; yet, resistance to chloramphenicol may also be associated with modification of the antibiotic target by chloramphenicol acetyltransferases encoded by *cat* genes (66). Moreover, the genes *sul1-3*, mediating resistance to sulphonamides, have been detected in all predominant serovars, while genes *dfrA* encoding resistance to trimethoprim were only detected in the monophasic variant I 4, [5], 12:i:–, Enteritidis, London, and Thompson. Furthermore, the genes *qnr* encoding decreased susceptibility to quinolones were detected in the monophasic variant I 4, [5], 12:i:–, Typhimurium, London, Derby, Anatum, Meleagridis, and Thompson. The resistance to quinolones could be due to the acquisition of resistance genes carried on mobile plasmids as well as the presence of chromosomic mutations affecting *gyr* and *par* genes (18, 67).

The virulome analysis of the studied isolates showed the detection of the typical genes implicated in the virulence and pathogenicity mechanisms of *Salmonella*. In addition, the gene *cdtB* encoding typhoid toxin production was detected in one isolate of *S*. Jangwani. In fact, *cdtB* gene has become commonly detected in NTS isolates from different sources (5, 12, 14, 18, 20, 23). It is noted that typhoid toxin was first found in *S*. Typhi and then several NTS serovars have been reported to carry genes encoding the production of this toxin, especially *cdtB*, suggesting the implication of this gene in the development of invasion non-typhoidal salmonellosis. Moreover, we reported the detection of *spv, pef*, and *rck* genes in isolates belonging to serovars Typhimurium and Enteritidis. The genes clustered in the *spv* locus have been demonstrated to possess an essential role in the virulence pathway of NTS (68, 69). The *pef* fimbrial operon mediates adhesion to the murine small intestine (70), while *rck* gene enhances bacterial adhesion and invasion and confers resistance to antimicrobial agents (71). On the other hand, *sodCI* confers resistance to oxidative burst inside macrophages (72, 73). Interestingly, these genes are carried on mobile genetic elements, including plasmids and phages, horizontally transferred between serovars, which may confer a virulence potential to new serovars that have not been reported to cause human gastroenteritis.

Mobile genetic elements are the leading factors for the high spread of antimicrobial resistance by transferring resistance and virulence genes between bacteria. In this study, we detected 18 different plasmids with the dominance of IncFIB(S) and IncFII(S). These plasmids may carry *pef* genes mediated adhesion to the murine on intestine (74), and *spv* genes necessary to the virulence pathway of NTS (75), in addition to their ability to confer hypervirulence and bacterial fitness (76). As known, the distribution of some plasmids is serovar dependent. In this study, we reported the detection of plasmids IncFIB(S) and IncFII(S) in *S*. Enteritidis and *S*. Typhimurium, while the plasmids IncHI2A and IncHI2 were only detected in *S*. I 4, [5], 12:i:–. Moreover, the association between plasmids and antimicrobial resistance genes showed that the plasmid type IncX1 is the leading carrier of the resistance genes *sul2, aph (3”)-Ib, aph (6)-Id, tet (A)* in *S*. Enteritdis. These results are in accord with those reported by Li et al. showing that the plasmid IncX1 is the key carrier of antimicrobial resistance determinants like *bla*_TEM−1B_, *sul1, sul2, aph (6)-Id, ant (3”)-Ia, aadA5, dfrA17, dfrA1*, and *tet(A)* in *S*. Enteritidis (18). Otherwise, Elbediwi et al. reported a strong positive correlation between the plasmids IncHI2 and IncHI2A and the resistance genes *sul1, dfrA12, armA*, and *bla*_TEM−1B_ (20). Therefore, the detection of *Salmonella* isolates harboring plasmids carrying virulence and resistance genes is a significant threat to public health.

## Conclusion

To date, few studies have investigated the epidemiological characteristics of NTS isolated from outpatients in China. Herein, we presented accurate information about the epidemiology of NTS implicated in human salmonellosis in Shaoxing city, Zhejiang province, China, by using whole-genome sequencing combined with in-depth bioinformatics analysis and cutting-edge phenotypic methods. Fifteen different serovars and 16 STs have been identified with the dominance of *S*. I 4, [5], 12:i:– ST34, *S*. Enteritidis ST11, and *S*. Typhimurium ST19, which were considered the leading cause of non-typhoidal salmonellosis in China and elsewhere. Interestingly, we showed that most isolates are MDR (83.91%) and harbor different antimicrobial genetic determinants carried on transferable plasmids, in addition to critical virulence genes implicated in the pathogenicity pathways of NTS. Hence the high incidence of MDR isolates in clinical NTS is a real issue for public health, threatening the current therapeutic procedures, which requires continuous monitoring of “superbugs” isolates using whole-genome sequencing.

## Data availability statement

The datasets presented in this study can be found in online repositories. The names of the repository/repositories and accession number(s) can be found at: https://www.ncbi.nlm.nih.gov/genbank/, PRJNA844576.

## Author contributions

JC and AE-D analyzed the data and finalized the figures. AE-D and MY wrote the manuscript. HZ and BW did the experiment and data collection. MY and YZ conceived the idea and assisted with data analysis and writing. All authors have read, revised, and approved the final manuscript.

## Funding

This work was supported by the National Program on Key Research Project of China (2019YFE0103900) as well as the European Union's Horizon 2020 Research and Innovation Programme under Grant Agreement No. 861917 – SAFFI, the National Natural Science Foundation of China (31872837 and 32150410374), Zhejiang Provincial Natural Science Foundation of China (LR19C180001), and Zhejiang Provincial Key R&D Program of China (2022C02024; 2021C02008; and 2020C02032).

## Conflict of interest

The authors declare that the research was conducted in the absence of any commercial or financial relationships that could be construed as a potential conflict of interest.

## Publisher's note

All claims expressed in this article are solely those of the authors and do not necessarily represent those of their affiliated organizations, or those of the publisher, the editors and the reviewers. Any product that may be evaluated in this article, or claim that may be made by its manufacturer, is not guaranteed or endorsed by the publisher.
